# Estimating directional epistasis

**DOI:** 10.3389/fgene.2014.00198

**Published:** 2014-07-14

**Authors:** Arnaud Le Rouzic

**Affiliations:** Centre National de la Recherche Scientifique, Laboratoire Évolution, Génomes, et Spéciation, UPR 9034Gif-sur-Yvette, France

**Keywords:** epistasis, genetic effects, estimation, statistics, evolution, multilinear model

## Abstract

Epistasis, i.e., the fact that gene effects depend on the genetic background, is a direct consequence of the complexity of genetic architectures. Despite this, most of the models used in evolutionary and quantitative genetics pay scant attention to genetic interactions. For instance, the traditional decomposition of genetic effects models epistasis as noise around the evolutionarily-relevant additive effects. Such an approach is only valid if it is assumed that there is no general pattern among interactions—a highly speculative scenario. Systematic interactions generate directional epistasis, which has major evolutionary consequences. In spite of its importance, directional epistasis is rarely measured or reported by quantitative geneticists, not only because its relevance is generally ignored, but also due to the lack of simple, operational, and accessible methods for its estimation. This paper describes conceptual and statistical tools that can be used to estimate directional epistasis from various kinds of data, including QTL mapping results, phenotype measurements in mutants, and artificial selection responses. As an illustration, I measured directional epistasis from a real-life example. I then discuss the interpretation of the estimates, showing how they can be used to draw meaningful biological inferences.

## 1. Introduction

An ability to understand and predict how genes affect morphological, physiological, and behavioral characteristics is of crucial importance in biology. This also poses a considerable challenge, given the complexity of the genetic architecture of quantitative traits (Flint and Mackay, 2009). This complexity is not only due to the large number of genetic, environmental, and physiological factors involved, but also to their multiple and nonlinear interactions. In particular, it was noticed very early in the history of genetics that the same genetic change often produces differing effects depending on the genetic background of the experimental species, population, or individual (Phillips, 1998; Wade et al., 2001; Phillips, 2008). The biological consequences of this phenomenon, known as “epistasis,” have triggered a considerable amount of discussion. A whole century of active research in genetics and molecular biology has revealed the ubiquity of epistatic interactions associated with the organization of biological systems as networks of interacting molecules (Omholt et al., 2000). However, we are still far from being able to integrate epistasis into a consensual, explicit, and predictive theoretical framework.

In the classical analysis of genetic variance (Fisher, 1918), epistasis is considered as a source of noise. Most epistatic effects are not transmitted from parent to offspring, and therefore, are not involved in the response to natural or artificial selection. Epistatic variance—the contribution of epistasis to genetic variance in a population—can be calculated (Cockerham, 1954; Kempthorne, 1954; Lynch and Walsh, 1998; Álvarez-Castro and Carlborg, 2007; Gjuvsland et al., 2007), but is almost meaningless in terms of predicting the genetic properties of a population (Barton and Turelli, 2004; Hansen, 2013; Álvarez-Castro and Le Rouzic, 2014), and may be negligible compared to evolutionarily-relevant additive genetic variance (Hill et al., 2008; Hemani et al., 2013).

Another idea, which has become popular only in recent decades, is that epistasis matters because of its capacity to affect additive variance rather than because of its contribution to interaction variance (Cheverud and Routman, 1995). In an epistatic genetic architecture, the effects of alleles on the phenotype depend on the genetic background. Accordingly, changes in the genetic background promoted by genetic drift (Goodnight, 1987, 1988; Barton and Turelli, 2004; Turelli and Barton, 2006; Álvarez-Castro et al., 2009; Jarvis and Cheverud, 2009) or by selection (Carter et al., 2005; Hansen et al., 2006; Hallander and Waldmann, 2007; Le Rouzic et al., 2013) may reveal, hide, or revert allelic effects, and thus significantly affect the genetic variance.

### 1.1. Directional epistasis

Epistasis can only exert a significant long-term influence on populations if individual epistatic effects do not tend to cancel out each other, i.e., if a general pattern emerges. The most obvious pattern is the directionality of epistasis, the fact that genetic interactions can be biased toward either high or low phenotype values. Estimates of directional epistasis allow to make useful predictions about the evolutionary potential of populations: if additive genetic variance is a measure of evolvability (Houle, 1992; Hansen et al., 2011), then the directionality of epistasis is a measure of genetic architecture asymmetry, i.e., how evolvability is influenced by the direction of evolution. When epistasis is positive, evolution is easier in the direction of high, rather than low, phenotypic values (because additive genetic variance tends to increase with the phenotypic value). In contrast, negative epistasis favors evolution toward low phenotypic values.

In spite of its predictive and descriptive value, directional epistasis is rarely reported for quantitative characters (Pavlicev et al., 2010). This can be attributed to two main factors: (i) many (if not most) quantitative geneticists are used to measuring epistasis via epistatic genetic variance, in spite of its marginal interest, and (ii) very few statistical or computational tools have been devised for measuring directional epistasis. The aim of this article is to present several methods for estimating directional epistasis from genetic and phenotypic data, and to propose accessible statistical procedures for computing epistasis. Several such methods will be illustrated from a real-life biological example, the genetic architecture of bodyweight in chicken, which displays a clear and consistent signal of positive epistasis. The data is based on a long-term artificial selection experiment on chicken body weight, and features (i) times series of the phenotypic response to selection, (ii) Quantitative Trait Locus (QTL) mapping data from a cross between the divergent lines, and (iii) minimal line-cross information (means of *F*_1_ and *F*_2_ populations) from the QTL setting.

### 1.2. Genetic models

In general, measuring the directionality of epistasis requires a model of genetic effects, i.e., a mathematical description of the relationships between the data (for instance, individual genotypes or phenotypes) and parameters to be estimated. The desirable properties for a “good” model of genetic effects depend on both the biological question and the nature of the data, and have resulted in rewarding (and sometimes conflictual) discussions (Cheverud and Routman, 1995; Hansen and Wagner, 2001b; Kao and Zeng, 2002; Yang, 2004; Zeng et al., 2005; Wang and Zeng, 2006; Álvarez-Castro and Carlborg, 2007; Aylor and Zeng, 2008; Hansen, 2014).

Genetic models can be conveniently divided into physiological and statistical models (Cheverud and Routman, 1995). In physiological (or functional: Hansen and Wagner, 2001b) models, genetic effects are described relative to a reference genotype, which can be arbitrary (for instance, one of the parental strains in an intercross) or conventional (typically, the wild genetic background). Functional models are generally rooted in traditional Mendelian genetics, in which a limited number of genotypes are experimentally generated and compared to reference strains. In contrast, statistical models quantify genetic effects in polymorphic populations across multiple genotypes. They are derived from the classical decomposition of genetic variance. Statistical genetic effects depend on allelic frequencies, and thus change when populations evolve; they provide a population-specific description of the genotype-to-phenotype map. In spite of obvious historical and conceptual divergences, it is sometimes possible to express both functional and statistical models in common mathematical frameworks, and to transform functional into statistical estimates (and *vice versa*) by means of “change of reference” operations (Hansen and Wagner, 2001b; Álvarez-Castro and Carlborg, 2007; Le Rouzic and Álvarez-Castro, 2008).

With respect to epistasis, another useful distinction can be made between unidimensional and multidimensional models (Kondrashov and Kondrashov, 2001; de Visser et al., 2011). Unidimensional epistasis describes the general curvature of the genotype-phenotype map, and can be interpreted as the average effect of allelic substitutions that would be observed if all loci were exchangeable. Multidimensional epistasis accounts for the complexity of the genotype-phenotype relationship, by characterizing all pairs of loci that have a specific epistatic effect. While directional epistasis is unidimensional by definition, it can be measured based on either unidimensional or multidimensional models.

Several models of directional epistasis will be reviewed below, starting from the multilinear model of epistasis, originally functional and multidimensional, which has been extended toward statistical and unidimensional formulations. I will then present and discuss alternative functional unidimensional models that are commonly used to measure epistasis for fitness, and show how they can be applied to quantitative characters.

## 2. Multilinear epistasis

### 2.1. The multilinear model of genetic interactions

#### 2.1.1. General framework

The multilinear model of genetic interactions developed by Hansen and Wagner (2001b) extends and makes explicit the concept of directional epistasis in quantitative genetics, and makes it possible to build genotype-to-phenotype maps implementing directional epistasis. In its original multidimensional form, the model expresses the phenotype *z* as a multilinear function of the genotype *G* of an individual. For two loci, labeled “1” and “2” respectively,

(1) $$z_{G}=z_{R}+y_{1_{R}}+y_{2_{R}}+y_{1_{R}}y_{2_{R}}\varepsilon_{12}.$$

Genetic effects are measured relative to an arbitrary reference genotype for which *y*_1_ = *y*_2_ = 0, associated with a reference phenotype *z*_*R*_. The effect of substituting the genotype of interest at locus 1 in the reference genotype *R* is *y*_1_*R*__, and conversely, *y*_2_*R*__ is the effect at locus 2. When introducing the genotype of interest at both loci, in the absence of epistasis, the phenotype is expected to change by *y*_1_*R*__ + *y*_2_*R*__. Any deviation from this expected additive outcome is attributable to epistasis. The originality of the multilinear model is to assume that this deviation is proportional to the product of allelic effects, the proportionality coefficient ε_12_ quantifying the strength and directionality of epistasis between loci 1 and 2.

**Figure 1:**
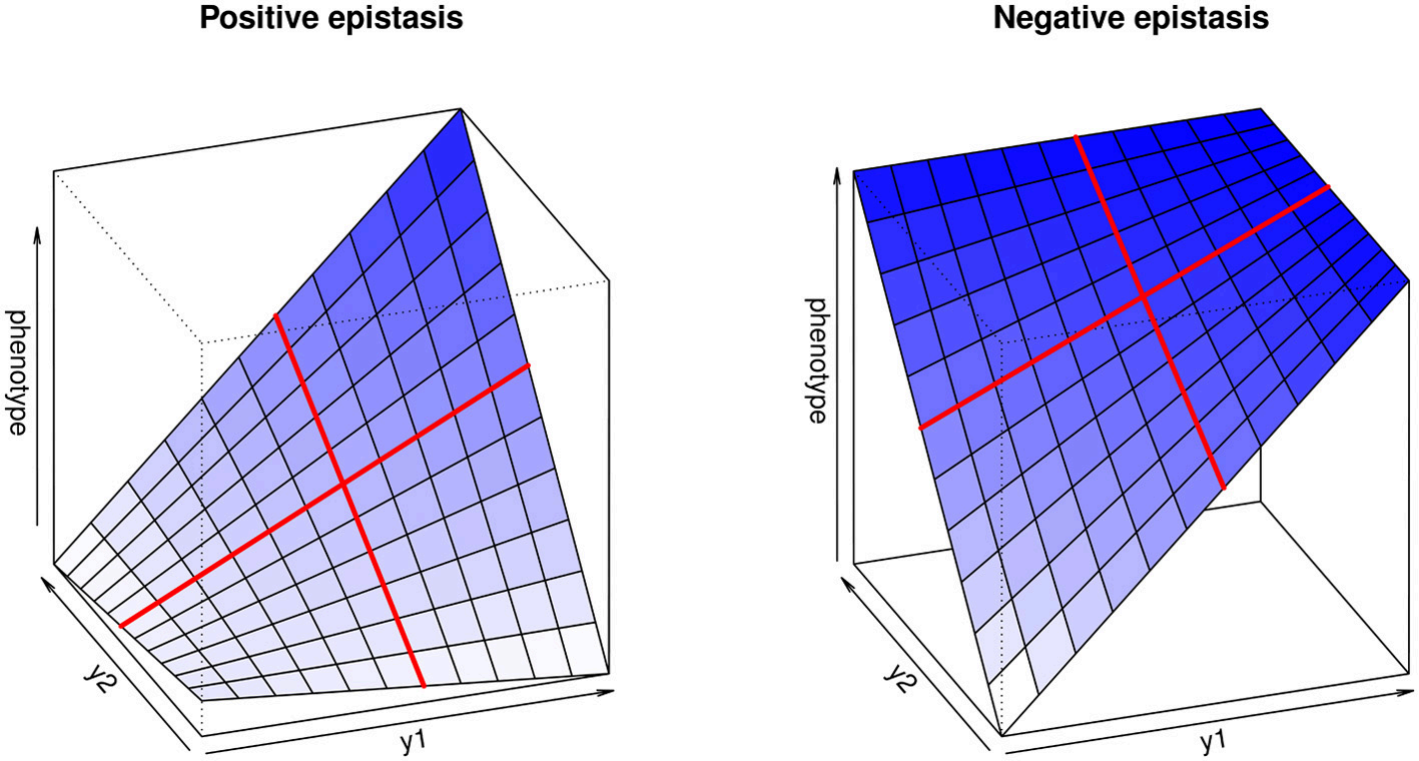
**Multilinear genotype-phenotype maps for two loci, illustrating positive (synergistic) and negative (antagonistic) epistasis**. *y*_1_ and *y*_2_ represent the genotype values at both loci. The red lines highlight the multilinearity of the model: if the genetic background is kept constant, phenotype change depends linearly on the genotype at each locus.

The multilinearity arises from the fact that any change in the genotype of a locus when keeping the genetic background constant leads to a proportional change in the phenotype. For instance, Equation (1) can be reformulated as *z*_*G*_ = *a* + *fy*_1_*R*__ (with *a* = *z*_*R*_ + *y*_2_*R*__ and *f* = 1 + *y*_2_*R*__ ε_12_), illustrating that the genotype-phenotype map is always linear with respect to single genotypes (Figure 1).

The epistatic coefficient, ε_12_, is expressed in terms of inversed phenotypic units (e.g., if the trait is measured in cm, ε will be in cm^−1^), which is not intuitive and does not allow comparisons between traits. Hansen and Wagner (2001b) suggest measuring epistasis by computing epistatic factors, *f*_1_ = 1 + *y*_2_ ε_12_ and *f*_2_ = 1 + *y*_1_ ε_12_, which quantify how much locus 1 is affected by locus 2, and *vice versa*; *f* = 1 implies no epistasis, *f* < 1 negative (antagonistic) epistasis, and *f* > 1 positive (synergistic) epistasis.

#### 2.1.2. Statistical formulation

The multilinear model is built as a functional model, since it defines genetic effects relative to a reference genotype, but a “change of reference” tool can be used to recompute genetic effects in any genotype or weighted combination of genotypes. When genetic effects are calculated relative to the average genotype of a population, the marginal contributions of individual loci coincide with additive effects, and the model can be considered to be statistical.

The multilinear model can also be used as a local approximation on a non-multilinear genotype-phenotype map. There are various ways of generating genotype-phenotype maps, which are multidimensional mathematical functions *g*(*y*_1_, *y*_2_, …, *y*_*n*_) that provide a deterministic phenotypic value for a series of genotypic values *y*_*i*_ at *n* loci. Such mathematical maps are often defined in theoretical work intended to explain the evolution of populations in complex genetic landscapes. Furthermore, even if the lack of large empirical genotype-phenotype data sets means that it is not yet realistic to attempt to do so, it is in principle possible to fit smooth surfaces (such as multidimensional splines) to experimental measurements, and thus generate models of genetic landscapes that could be analyzed mathematically (and tested empirically).

**Figure 2:**
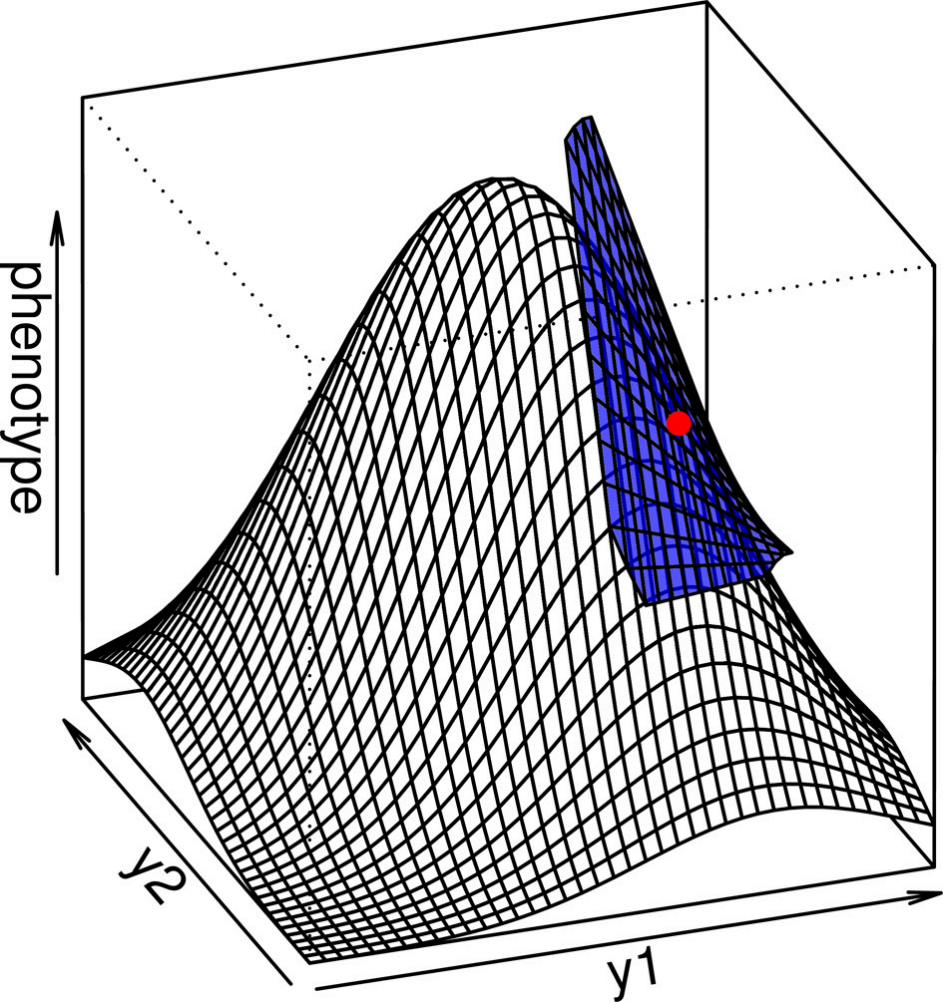
**The multilinear model (blue surface) is a local approximation of the interlocus curvature in a complex genotype-phenotype map**. When the average genotype is chosen as the reference (red point), the multilinear approximation is able to predict the evolutionary properties of the population in a more precise way than the additive model.

In any case, the multidimensional directional epistasis coefficients ε_*ij*_, which measures the curvature of the genotype-phenotype function between loci *i* and *j*, can be directly quantified as ε_*ij*_ = *D*^2^_*ij*_/*D*_*i*_ *D*_*j*_, where *D*_*i*_ = ∂ *g* / ∂ *y*_*i*_ is the value of the first partial derivative of function *g* taken at the reference point, and *D*^2^_*ij*_ = ∂^2^*g*/∂ *y*_*i*_ ∂ *y*_*j*_ is the mixed partial derivative (the curvature of the function *g* across both loci). This result illustrates the fact that the multilinear model is similar to a Taylor expansion of the genotype-phenotype map that ignores intra-locus curvature (Hansen and Wagner, 2001b) (see Appendix I and Figure 2).

#### 2.1.3. Composite directional epistasis

The original multilinear model is multidimensional, as it involves as many ε_*ij*_ parameters as pairs of loci. A unidimensional (and statistical) version of the model was proposed in Carter et al. (2005), with the composite directional epistasis coefficient ε_*c*_ calculated as the average ε_*ij*_ coefficient weighted by the additive genetic variance explained by each pair of loci:

(2) $$\varepsilon_{c}=\frac{\sum_{i}\sum_{j\;\neq \;i}V_{A_{i}}V_{A_{j}}\varepsilon_{ij}}{\sum_{i}\sum_{j\;\neq \;i}V_{A_{i}}V_{A_{j}}}.$$

Both uni- and multi-dimensional versions of the model can be extended to higher orders of interactions and to multiple traits (Hansen and Wagner, 2001b).

### 2.2. Directional epistasis from phenotypic data

#### 2.2.1. Response to artificial selection

Directional epistasis affects evolution, as it changes the amount of genetic variation available depending on the direction of phenotypic change (Hansen et al., 2006). For instance, selection in the direction of positive epistasis tends to increase the frequency of synergistic genetic interactions, thus enhancing the effect of selection. In contrast, selection in an antagonistic system decreases the genetic variance, and thus decreases the selection response. These effects can be experimentally observed, especially with bidirectional artificial selection responses, since they are expected to generate asymmetric responses in up- and down-selected lines.

***2.2.1.1. Theoretical framework.*** It is possible to model the expected impact of directional epistasis on genetic variance and to predict the difference between up- and down-selected lines as a function of the epistatic coefficients. Using a series of simplifying assumptions detailed in Appendix II, the selection response under a constant selection gradient after *t* generations is expected to be:

(3) $$\begin{array}{l} \mu_{t}≃\mu_{0}-\frac{\log(1-2\Delta_{\mu_{0}}\varepsilon t)}{2\varepsilon }\\ \;\approx \mu_{0}+\Delta_{\mu_{0}}t+\varepsilon \Delta_{\mu_{0}}^{2}t^{2}+\ldots , \end{array}$$

where μ_0_ is the initial mean phenotype, Δ_μ_0__ is the initial selection response (after the first generation), and ε is the directionality of epistasis. The second part of the equation is the second-order Taylor approximation around *t* = 0, illustrating the linear selection response expected by the traditional breeder's equation (Δ_μ_0__ *t*), and how directional epistasis appears as a quadratic term. Here, ε is the unidimensional directional epistasis, and thus corresponds to ε_*c*_ in Equation (2).

**Figure 3:**
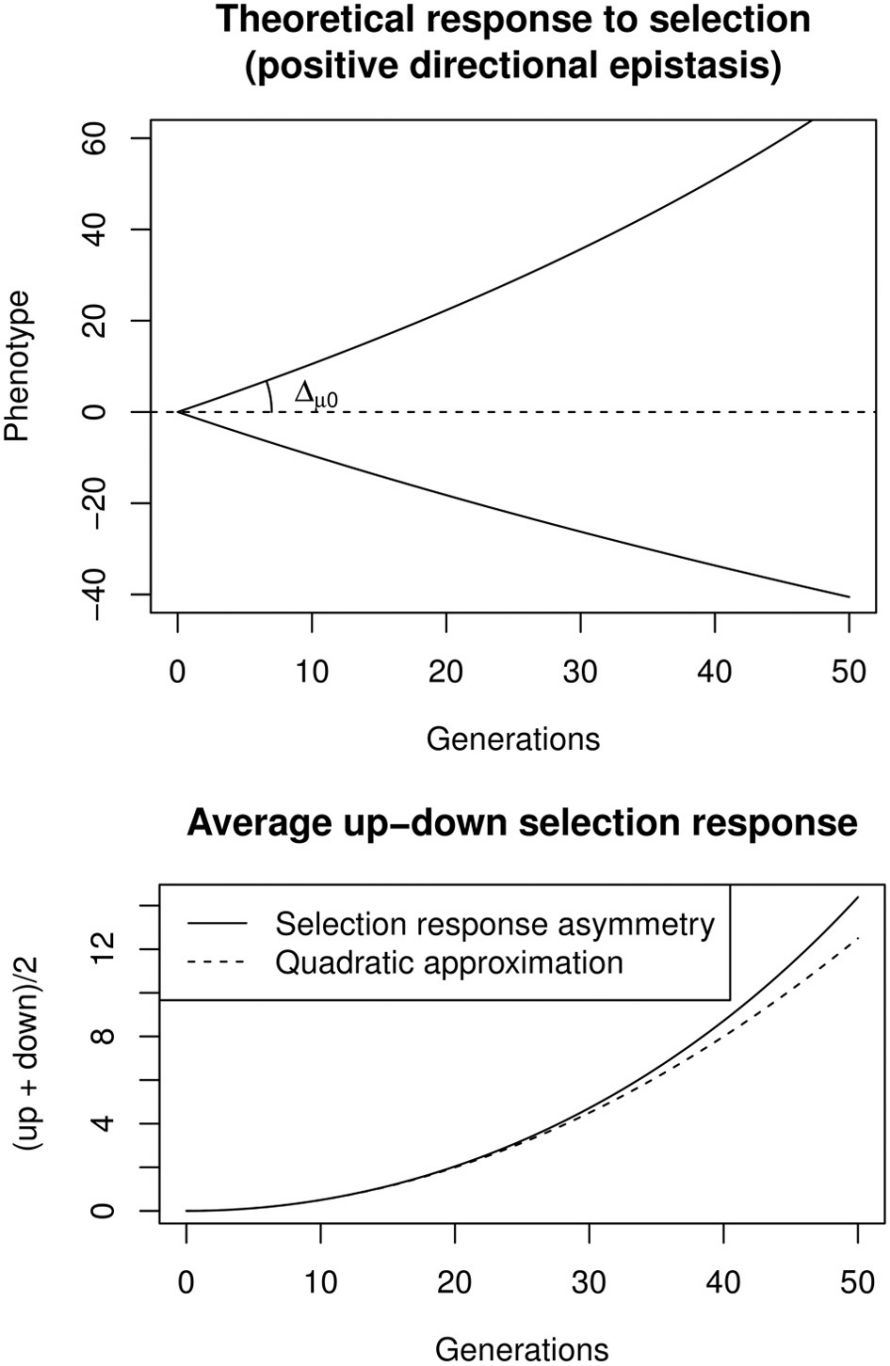
**Top:** Theoretical response to bidirectional constant selection under positive directional epistasis (ε = + 0.005, Δ_μ0_ = 1). **Bottom:** the selection response is asymmetric, and the up-down average increases almost quadratically with time, the quadratic coefficient being ε Δ^2^_μ0_.

A convenient way to estimate directional epistasis from bidirectional selection responses is to compute the up/down asymmetry through the average selection response, *A*(*t*) = $\frac{1}{2}$ (up(*t*) + down(*t*)) (Figure 3). If epistasis is directional and relatively weak (Δ_μ_0__ ε ≪ 1), *A*(*t*) changes approximately with *t*^2^, such that *A*(*t*) ≃ ε Δ^2^_μ0_ *t*^2^. It is thus possible to estimate Δ_μ_0__ as the slope at origin of the selection response, and then ε through a quadratic regression on the average up/down response. Including the effects of e.g., inbreeding, linkage disequilibrium, or canalization, is possible, but requires to numerically maximize the likelihood of complex models. This can be done with the software package sra for R, described in Le Rouzic et al. (2011).

***2.2.1.2. Example: artificial selection on body weight.*** For more than 50 years, two chicken (*Gallus gallus*) lines were selected for high and low body weight at 56 days, respectively (Siegel, 1962; Liu et al., 1994; Dunnington and Siegel, 1996). The experiment is still ongoing; here, I consider the latest phenotypic results available (54 generations, Dunnington et al., 2013). For simplicity, only the time series of mean phenotypes are considered, although some variance estimates were also available in this case.

**Figure 4:**
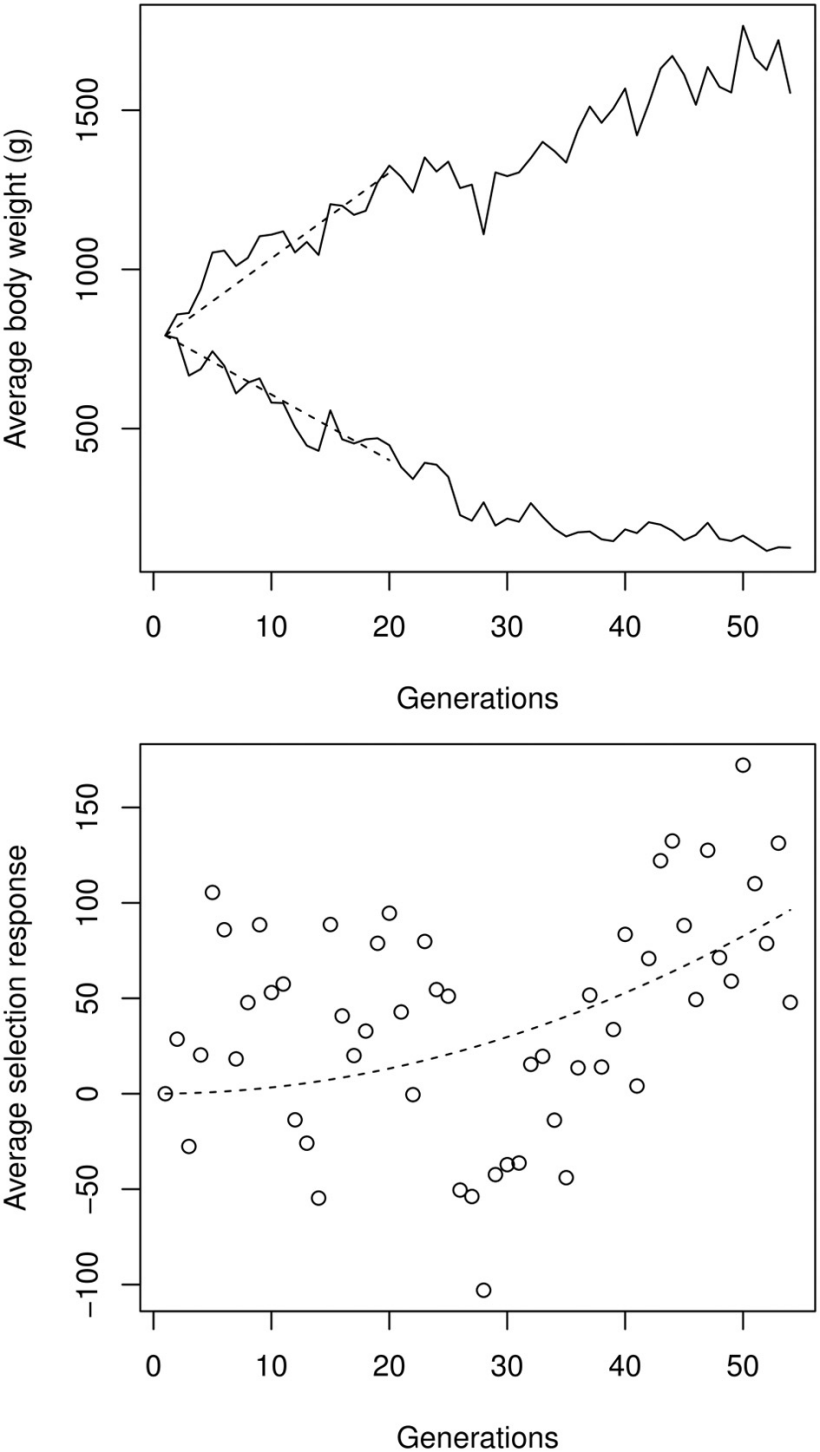
**Top:** male-female average experimental selection response on chicken bodyweight, digitalized from Figures 1, 2 in Dunnington et al. (2013). The initial selection response Δ_μ_0__, estimated by a linear regression over the first 20 generations (dashed segments), was 25.6 g per generation in the high line, and −19.6 g per generation in the low line. **Bottom:** quadratic regression on the up- and down-selection average, illustrating the cumulative effect of directional epistasis. The quadratic coefficient (which is an approximation of Δ^2^_μ0_ε), estimated by a non-linear, least-square regression, was 0.033 g per generation squared.

The impact of artificial selection was considerable (Figure 4). In the high-selection line, the body weight at 8 weeks rose from 800 g (male-female average) to 1650 g. In the low-selected line, the average body weight decreased to around 150 g, leading to an impressive order-of-magnitude difference between high- and low-selected lines, well beyond the differences usually observed between closely-related species, and spanning more than one third of the relative weight diversity in the entire 20 Myr-old Galliformes order. The selection response was asymmetric: although the selection strength was identical in both lines, progress was slower in the low line. This can easily be attributed to epistasis, given the expected differences in the genetic backgrounds of 1500 vs. 150 g birds.

Using the procedure described in Equation (3), the strength of directional epistasis could be estimated from a quadratic regression over the high-low asymmetry. Estimating the initial selection response at around |Δ_μ_0__| = 22.6 g per generation on average, directional epistasis is ε ≃ + 6.6 × 10^−5^ g^−1^. Although apparently small, this figure is statistically significant and generates cumulative effects on genetic architectures: Any phenotypic change corresponding to the initial (first-generation) selection response induces an increase of allelic effects of 0.15% in the high line, and decreased accordingly in the low line. The same allele is thus expected to display a >10% difference in the two extreme genetic backgrounds, representing weak, but non-negligible, epistasis.

Of course, this estimate relies on major assumptions about the underlying process. Several genetic or non-genetic factors other than epistasis could affect the available genetic variance, and thus bias ε. For instance, the quadratic approximation relies on the hypothesis that the selection gradient is constant over the entire time series, whereas in fact we know from e.g., Dunnington et al. (2013) that the selection intensity actually increases with time. Meanwhile, the reduced population size in the experiment necessarily generated a significant amount of inbreeding (even with a carefully-designed breeding scheme), which decreases the variance due to genetic drift. However, these mechanisms are unlikely to generate misleading estimates of ε, since (i) they affect both the up and down lines in the same way, and so cannot generate any asymmetry, and (ii) they tend to offset each other, as the selection strength increases while the genetic variance decreases.

More worrisome is the possibility of uncontrolled natural selection in the low line. A fraction of the smallest birds appeared to be sterile or unviable, which could contribute to the slowing-down of the response. Such a mechanism could generate an asymmetric response, and thus spurious positive estimates of the epistatic coefficient. Nevertheless, this seems rather unlikely, given the behavior of the twelve relaxed selection lines presented in Dunnington et al. (2013). Indeed, when selection was stopped in both lines, the populations did not tend to evolve back to the original phenotype, as would have been expected if natural selection was preventing the population from responding to artificial selection. The phenotypic data therefore seems to be compatible with a genetically-driven asymmetry, due to smaller allelic effects in low-weight chickens (i.e., positive epistasis).

#### 2.2.2. Line-cross analysis

With the improvement in sequencing and genotyping technologies, the phenotype-based methods developed and used by quantitative geneticists for most of the 20th century to investigate genetic architectures without resorting to genotype data are currently losing popularity. However, they are still both elegant and informative, especially when used to estimate general properies of populations such as unidimensional directional epistasis. One of the most powerful (and simple) of these biometric methods consists of crossing individuals or strains of interest in order to generate hybrid and backcross populations, from which the phenotypic means and variances can be determined. The knowledge of the transmission mechanisms of genetic factors from parents to offspring makes it possible to disentangle the impact of additive, dominance, and epistatic effects on the genetic differences between the original individuals (Lynch and Walsh, 1998 p. 205).

A set of equations that can be used to compute additive, dominance, and directional epistatic effects from parental, intercross, and backcross populations are provided in Hansen and Wagner (2001b) (see Demuth and Wade, 2005, for an alternative model). Directional epistasis is unidimensional, and thus corresponds to the ε_*c*_ parameter of Equation (2). Below, a slightly different parameterization will be used, in which both parental populations are separated by four additive effects, so that the model is identical to a 2-locus QTL effect model in a diploid species. The model was set up so that genetic effects cancel out in the *F*_2_ population, but a different reference point can be chosen (using the genetic effect matrices provided in, e.g., Álvarez-Castro and Carlborg, 2007). Average phenotypes for both parental populations (*P*_1_ and *P*_2_) and the first two intercross populations *F*_1_ and *F*_2_ can be express as functions of four parameters: a reference μ (arbitrarily, the mean *F*_2_), additive and dominance effects *A* and *D*, and the directional epistasis coefficient ε.

(4) $$\begin{array}{l} P_{1}=\mu -2A-D+\varepsilon (A^{2}+AD+\frac{1}{4}D^{2})\\ P_{2}=\mu +2A-D+\varepsilon (A^{2}-AD+\frac{1}{4}D^{2})\\ F_{1}=\mu +D+\frac{1}{4}\varepsilon D^{2}\\ F_{2}\;=\mu . \end{array}$$

This simple model can be illustrated by the data from the experimental cross between the two chicken strains (Dunnington and Siegel, 1996; Marquez et al., 2010). In this experiment, the two generations of crossing necessary to generate a polymorphic F_2_ population for QTL mapping makes it possible to sketch a minimal line-cross analysis. Both parental populations as well as F_1_ and F_2_ individuals were raised in the same location, with the same food, and at the same density; their average weights at 8 weeks were 170 and 1412 for both parental chicken populations respectively, 650 g for the F_1_, and 624 g for the F_2_. Both F_1_ and F_2_ are below the parental arithmetic average (791 g), suggesting the presence of dominance and/or epistatic effects (Álvarez-Castro et al., 2012).

Although not perfect, this setting makes it possible to estimate up to four genetic parameters. Two models, with and without dominance, were tested, and gave very similar results (Equation 4 and Table 1). The dominance effect, when estimated, was an order of magnitude below the additive contribution. Epistasis was positive, and of similar magnitude in both models.

**Table 1:** **Epistatic line-cross analysis of the chicken lines**.

**Effect**	**No dominance**	**Dominance**
Reference μ	637 g	624 g
Additive *A*	310 g	318 g
Dominance *D*	-	26 g
Directional epistasis ε	1.6 × 10^−3^ g^−1^	1.9 × 10^−3^ g^−1^

The full model (involving dominance) has no degree of freedom, so that statistical errors cannot be estimated.

### 2.3. Directional epistasis from QTL data

Nowadays, data sets often consist of individuals in which both the phenotype and the genotype at loci of interest are known. This is for instance the case after the mapping of Quantitative Trait Loci (QTLs), either by linkage or association methods. Such data sets represent a valuable source of information about epistasis, and in particular about multidimensional epistasis, which can hardly be estimated from phenotypic data.

#### 2.3.1. Linear and multilinear models of genetic effects

In most cases, QTL mapping procedures only focus on marginal (additive and dominance) effects, and do not explicitly consider genetic interactions (Carlborg and Haley, 2004). However, epistasis may be of major interest, both for improving QTL detection (Carlborg et al., 2003, 2004, 2006), and for the biological interpretation of the genotype-phenotype relationship (Malmberg and Mauricio, 2005; Le Rouzic et al., 2007, 2008). Mapping procedures accounting for epistasis generally rely on components of the interaction variance (Cockerham, 1954; Kempthorne, 1954; Lynch and Walsh, 1998), which makes it necessary to estimate four genetic effects for each pair of loci (additive-by-additive, additive-by-dominant, dominant-by-additive, and dominant-by-dominant statistical effects). More recently, “variance QTL” approaches have been proposed to map loci involved in various kinds of interactions, including gene-gene and gene-environment interactions (Rönnegård and Valdar, 2012). Until recently, there was no QTL mapping method based on directional epistasis (Slatkin and Kirkpatrick, 2012), and estimation from genotype-phenotype data usually relied on model fitting on a predefined set of candidate loci (Cheverud et al., 2001; Le Rouzic et al., 2008; Shao et al., 2008; Pavlicev et al., 2010; Jarvis and Cheverud, 2011).

The traditional genetic regression model, ignoring dominance (and dominance-related epistatic components), can be written as:

(5) $$P_{y_{1},y_{2}}=\mu +\alpha_{1}S_{1}+\;\alpha_{2}S_{2}+\;\alpha \alpha_{12}S_{12}.$$

This model has 4 parameters for a pair of loci: μ is the intercept of the model (reference point), α_1_ and α_2_ are the additive effects for both loci, and αα_12_ — a traditional (and probably unfortunate) notation, not to be confused with the product α × α_12_ — is the additive-by-additive effect. The *S* coefficients determine the genetic model, i.e., the weights of the genetic effects for each genotype. For instance, consider a haploid two-locus two-allele system with the reference genotype (arbitrarily) set to *A*_1_ *B*_1_. In the reference genotype, all *S* coefficients are set to 0 (μ, the reference point, thus corresponds to the intercept of the model). For genotype *A*_1_ *B*_2_, *S*_1_ = 0, *S*_2_ = 1 (because 1 effect α_2_ has been added to the model, given the substitution of a *B*_2_ allele), and *S*_12_ = 0. In genotype *A*_2_ *B*_2_, *S*_1_ = 1, *S*_2_ = 1, and *S*_12_ = 1, reflecting the possibility of an interaction between *A*_2_ and *B*_2_ alleles. Of course, different reference points can be chosen, including mixtures of genotypes in specific frequencies (such as in the *F*_2_ model, considering even allelic frequencies and Hardy-Weinberg proportions). The models becomes more complex with diploid genotypes (which include dominance effects), but the principle remains the same. Below, I used the model “NOIA” proposed by Álvarez-Castro and Carlborg (2007), which has some interesting statistical features. In particular, the model is orthogonal (provided there is no linkage disequilibrium) even if the population is not at Hardy-Weinberg proportions. In “NOIA,” the *S* coefficients are stored as a genetic design matrix, and the model can be extended (to include more alleles and/or more loci) using simple matrix algebra.

It is possible to modify the above framework to estimate directional epistasis. The strategy proposed by Le Rouzic and Álvarez-Castro (2008) is based on a non-linear, least-square regression, very similar to the framework proposed in Equation (4) for the analysis of line crosses: the model explicitly decomposes the epistatic parameter as a multilinear combination of additive effects, assuming that αα_*ij*_ = α_*i*_ × α_*j*_ × ε_*ij*_:

(6) $$P_{y_{1},y_{2}}=\mu +\alpha_{1}S_{1}+\;\alpha_{2}S_{2}+\;\alpha_{1}\alpha_{2}\varepsilon_{12}S_{12}.$$

This setting can easily be extended to account for dominance and higher-order epistasis (Álvarez-Castro and Carlborg, 2007; Le Rouzic and Álvarez-Castro, 2008; Pavlicev et al., 2010). When ε_*ij*_ is estimated for each pair of loci, the model describes multidimensional epistasis. There are two distinct ways to estimate unidirectional epistasis from this setting. The first method is to assume that ε is identical between loci, i.e., replacing ε_*ij*_ by a constant ε in Equation (6). The second strategy is to estimate independent ε_*ij*_ values for each pair of loci, and to compute the composite epistasis ε_*c*_ using Equation (2). This last strategy is more theoretically-grounded than the former, but it rapidly becomes impractical when the number of loci increases: the number of interactions increases quadratically with the number of loci, which reduces the precision of pairwise interaction estimates.

#### 2.3.2. Application to QTLs for body weight

Individuals from both the high and low chicken lines were intercrossed at generation 46, to form the F_1_ and F_2_ populations described above. The 795 surviving individuals from the F_2_ population were phenotyped for various characters and genotyped for 145 genetic markers on 25 chromosomes. The QTL mapping analysis identified 6 significant loci (four major loci and two of lesser effect). These significant loci combined explained around 10% of the phenotypic variance, and strong epistatic interactions have been reported among them (Carlborg et al., 2006; Le Rouzic et al., 2007; Álvarez-Castro et al., 2012). For the sake of both simplicity and statistical power, only the four major QTLs are considered in the subsequent analyses.

There are 24 second-order epistatic interactions between four loci (6 additive-by-additive, 6 dominance-by-dominance, and 12 additive-by-dominance interactions). It is possible to estimate all of them using a model performing the traditional decomposition of genetic effects (here, I used the software package noia for R, Le Rouzic and Álvarez-Castro, 2008), but interpreting these 24 independent epistatic estimates is complicated: in spite of the large sample size (around 800 individuals), only 4 (out of 24) epistatic estimates reached the 5% *p*-value threshold, and none remained statistically significant after correction for multiple-testing. There were no obvious signs of directional epistasis (11 positive estimates out of 24), even when focusing on additive-by-additive epistasis (3 positive estimates out of 6).

Fitting a unidimensional multilinear model of epistasis leads to a much more conclusive analysis. The estimated constant ε coefficient is positive (ε = +0.057 g^−1^). The weighted composite parameter, calculated from Equation (2), is also positive and of the same order of magnitude (ε_*c*_ = +0.020 g^−1^). The multilinear model fits better than the traditional genetic-effects model with pairwise epistasis, outperforming it by 13.5 AIC units (ΔAIC scores >10 can be considered to be conclusive, Burnham and Anderson, 2002). The multilinear model is also considerably better than models without epistasis (ΔAIC = 18.5). The undisputable statistical superiority of the multilinear model translates into a substantial gain in explanatory power: the four-locus model without epistasis explains only 5.4% of the total phenotypic variance, while the multilinear model explains 7.8%.

## 3. Regressions against the number of mutations

While it is particularly rare to find estimates of directional epistasis for quantitative characters in general (Pavlicev et al., 2010), the sign of epistasis has been frequently estimated for fitness. The importance of directional epistasis for the logarithm of fitness has now been fully acknowledged by evolutionary biologists, as it affects the evolution of sex, recombination, mutation rates, and other related phenomena (Phillips et al., 2000). Here I will review two models frequently used in this context, and show how they can be modified to fit other quantitative traits. According to the previous definitions, these models are both functional and unidimensional, as they estimate directional epistasis with reference to the “wild type” with no mutations.

### 3.1. Model description

A common way to estimate directional epistasis for (log) fitness is a “power” (or “multiplicative”) model *W* = α *n*^β^ (illustrated in **Figure 6**), where *W* stands for the log-fitness, α is the effect of a single mutation, *n* is the number of mutations, and β measures directional epistasis. The model is based on the fact that the fitness of the reference individual or strain (*n* = 0) is 1, so that the intercept of the model is log(1) = 0 by construction. Fitness in single mutants (*n* = 1) is not affected by epistasis, which makes it possible to estimate α. Epistasis appears for *n* ≥ 2, generating deviations from linearity. β > 1 represents positive epistasis, while β < 1 stands for negative epistasis. The parameters of the model are usually estimated through non-linear regressions (least squares) or by non-linear generalized model approaches (maximum likelihood).

An alternative setting is the quadratic model *W* = −(α *n* + $\frac{1}{2}$ β′ *n*^2^) (Elena and Lenski, 1997; Kouyos et al., 2007) (for consistency with the literature, I have retained the same notation, although it should be noted that β and β′ have different units, and β′ > 0 means positive epistasis). This latter model has some interesting theoretical properties associated with the Gaussian fitness function, and is more firmly grounded in classical population genetics theory (Charlesworth, 1990; Otto, 2007).

Alternative parameterizations of the above models appear in the literature (e.g., estimating −α instead of α, or β −1 instead of β, which provides a more straightforward interpretation of “positive” and “negative” epistasis). This framework is generally used in two different experimental contexts: estimating the directionality of deleterious mutations (in which case, α < 0, and negative epistasis means that the deleterious mutations act synergistically to decrease fitness), or estimating epistasis among the beneficial mutations accumulated during an artificial evolution experiment (α > 0, and negative epistasis represents the antagonistic effects of mutations) (Lenski et al., 1999; Wilke and Adami, 2001; Maisnier-Patin et al., 2005). These symetric interpretations are arguably confusing, and the literature is not always consistent with regard to the association between the sign of directional epistasis and the synergistic or antagonistic properties of mutations (e.g., Szathmáry, 1993).

### 3.2. Model fitting

These models are clearly not suited for fitting traditional quantitative genetics data, in which there are no “wild type” or “mutants.” However, it is still possible to define the following continuous function for a phenotype *P*, which behaves in a similar fashion as the power model:

(7) $$P(m)\;=\;\{\begin{array}{ll} \mu +\alpha m^{\beta }, & \text{ if }m>0\\ \mu , & \text{ if }m=0\\ \mu -\alpha |m{|}^{1/\beta }, & \text{ if }m<0, \end{array}$$

where *m* is a real number analogous to the “number of mutations” compared to the reference genotype, α and β have the same meaning as in the power model (α is the average effect of the first mutation, and β is the epistatic coefficient, with β = 1 standing for no epistasis). μ is the intercept of the model, i.e., the phenotype of the “reference genotype.” This function is not differentiable at *m* = 0, but this is unlikely to affect the estimates. In order to obtain a proper analogy with traditional quantitative genetics, the mean F_2_ (same number of alleles from both parental lines) was chosen as the reference. *m*, the “number of mutations” parameter, thus stands for the number of additional “high-line” (H) alleles in a genotype compared to the reference. Considering the 4 significant QTLs, *m* = 0 for the reference (mean F_2_) genotype (which has 4 low-line alleles and 4 high-line alleles), *m* = −4 in the full low-line genotype (8 alleles from the low-line), and *m* = +4 in the full high-line genotype. An equivalent formulation (*P*(*m*) = μ + α *m* + $\frac{1}{2}$ β′ *m*^2^) can also be defined for the quadratic model.

**Figure 5:**
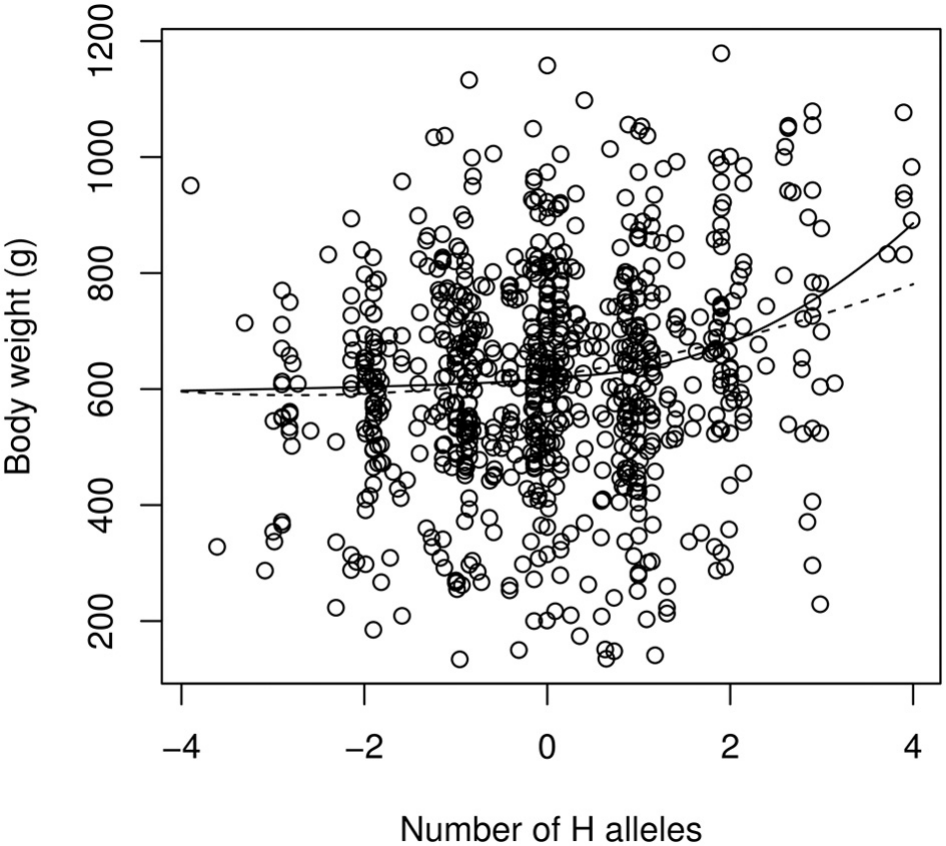
**The continuous version of the power model (Equation 7, solid line) and the quadratic model of epistasis (dashed line) applied to the chicken QTL data**. The reference genotype contains as many “low” (L) alleles as “high” (H) alleles. The x-axis scales from −4 (LL genotype at all loci) to +4 (HH genotype at all loci). Intermediate numbers of mutations are due to genotype uncertainties when QTLs are not in total linkage disequilibrium with markers.

Fitting the “continuous power model” of Equation (7) to the data by a non-linear, least-square procedure leads to the following estimates (estimate ± std. err.): α = 13.0 ± 5.8 g; β = 2.18 ± 0.41 (Figure 5). This is indicative of strong (and statistically significant) positive epistasis. The first allelic substitution in the reference background (average F_2_ individual) is thus expected to have an effect of 13 g, the second substitution will affect the phenotype by 45.9 g (two “high” substitutions) or 4.9 g (two “low” substitutions). The epistatic effect is extreme for the fourth substitution, which is predicted to have an effect of 124 g in the “high” direction (i.e., 10 times the estimated effect in the average genetic background) but only 3 g in the “low” direction. The estimate of directional epistasis in the power model is heavily influenced by the few “extreme” genotypes: the 7 individuals with eight “H” alleles are all far above the average, which contributes to the excessive curvature of the genotype-phenotype relationship (Figure 5). Yet, epistasis is still present when all extreme genotypes (full homozygotes LL and HH) are removed, with an estimate of β = 1.83 ± 0.50.

Estimates from the quadratic model are α = 23.1 ± 4.7 g, and β′ = 8.3 ± 4.0 g. In spite of the similar notation, β′ is not on the same scale as β, and directional epistasis, although significantly positive, is smaller here (the two first allelic substitutions in the direction of higher phenotypes have an effect of 27.3 and 35.6 g respectively, vs. 19.0 g and 10.7 g for one and two substitutions toward lower phenotypes).

## 4. Discussion

### 4.1. Model comparisons

Although they all provide an estimate of unidimensional directional epistasis, the models reviewed in this paper have been designed to address different questions, and based in different sub-fields of population and quantitative genetics.

The multilinear model provides an explicit description of epistasis between a set of loci, as in classical quantitative genetics models, and can be extended to fit to phenotypic data. On the opposite, both “regression” models suppose that epistatic patterns follow a general function. This incompatibility between models of directional epistasis for fitness and traditional quantitative genetics models is probably an important factor in the lack of experimental measurements of directional epistasis for quantitative traits (Hansen and Wagner, 2001a; Pavlicev et al., 2010).

**Figure 6:**
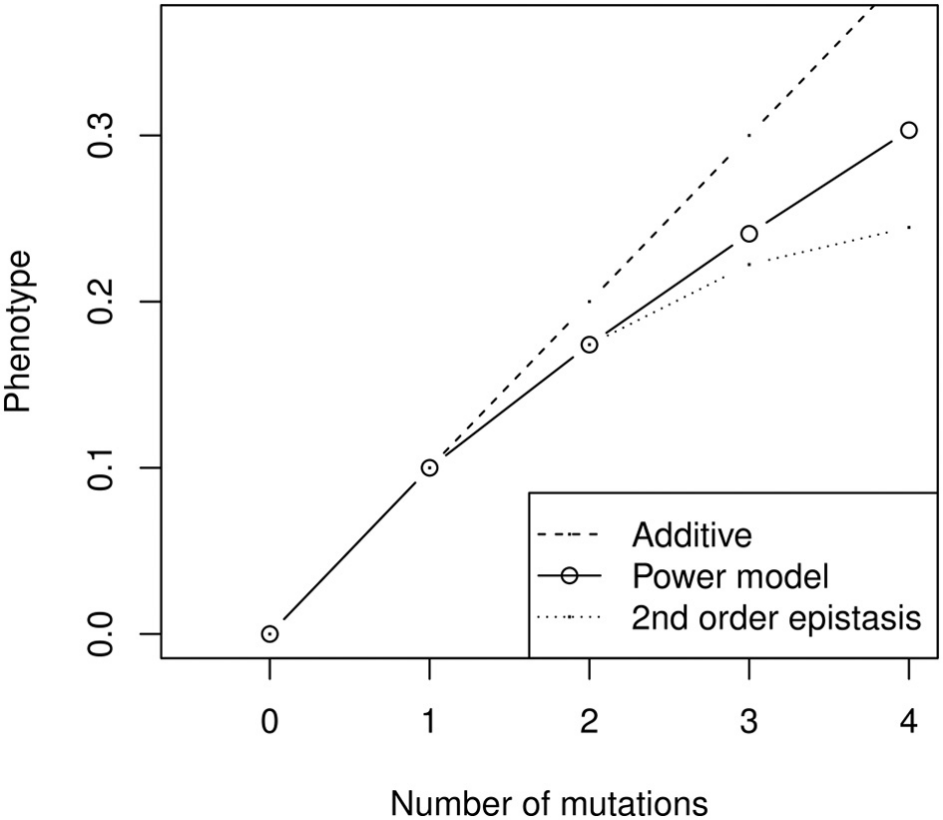
**Illustration of high-order epistatic effects in the power model (here with negative epistasis, α *n*^β^ with α = 0.1 and β = 0.8)**. The second-order epistatic effect is negative (the power model is always below the additive prediction), but the third-order effect is positive (the power model is always above the quadratic model). The sign of the interactions thus alternates when β < 2, and their relative size does not decrease rapidly. As a result, the effect of combining several mutants cannot be properly inferred from simpler combinations—for instance, the prediction for four mutants is not much better for the second-order epistatic model than for the additive model, and can even be worse with more substitutions.

In addition to the fact that models are not designed to be applied to the same kind of data (the need to compare genotypes to an arbitrary wild type or the assumption of constant mutational effect size are difficult to overcome for quantitative genetics data), models also carry conceptual differences about the nature of epistatic interactions. For instance, the power model necessarily involves highly complex epistatic interactions (Hansen and Wagner, 2001a). Quantitative genetics rely on linear models of genetic effects, in which interactions are calculated iteratively as the deviation between mutant phenotypes and the sum of lower effect interactions. The multilinear model follows this tradition, and is built as a sum of effects involving one locus (marginal effects), two loci (pairwise interaction effects), three loci, etc. For instance, second-order epistasis is the difference between the double mutant and twice the single mutant effect (Figure 6). In contrast, in the power model, there are as many interaction effects as there are mutations, which leads to very complex epistasis. For most realistic values of β (0 < β < 2), the second- and third-order interactions have opposite effects—in other words, if combining two mutations has antagonistic effects, combining three of them will have synergistic effects (the triple mutant is closer to additivity than predicted by the sum of second-order interactions). Moreover, the magnitude of high-order epistatic effects can represent a substantial fraction of lower-order effects (Figure 6), suggesting that combined mutant phenotypes are heavily impacted by the emergent properties of specific combinations of allelic substitutions, and thus difficult to predict from experimental results.

This issue is avoided with the quadratic model, which is limited to interactions between pairs of loci. However, this quadratic model implies that mutational effects can switch signs depending on the genetic background (sign epistasis). This property, which is sometimes perceived as undesirable when considering epistasis for fitness (Wilke and Adami, 2001), could explain the persistence of alternative models. Another side effect of most unidimensional models of epistasis for fitness is that mutations are assumed to be of constant size. Relaxing this assumption significantly alters the evolutionary properties of the system (Butcher, 1995; Otto and Feldman, 1997), casting doubts on the operational meaning of β (or β′) parameters.

### 4.2. Full-genome epistasis

For most of the 20th century, the concept of genotype-to-phenotype map was mostly virtual, and mainly used for theoretical purposes. The possibility to access complete individual genomes for a reasonable price has not really been anticipated by quantitative geneticists, and we are now in the uncomfortable situation of not being able to properly translate the massive amount of data collected experimentally into ground-breaking theoretical insights. Indeed, it is widely acknowledged that the revolutionary improvement in the quality and quantity of genotypic information has not generated a proportional improvement in our ability to describe the genetic architecture of quantitative traits from genome-wide association studies. This “missing heritability” problem might be partly due to our inability to detect properly epistatic interactions (Maher, 2008; Zuk et al., 2012; Hemani et al., 2013).

Identifying interacting pairs of loci from a genotype-phenotype dataset schematically follows two strategies: (i) combine epistatic and marginal effects while mapping loci, with the hope to increase the genetic signal (Carlborg and Haley, 2004), or (ii) first map loci based on their marginal effects, and estimate epistasis *a posteriori* between pairs of significant loci. Although theoretically elegant, the first strategy generally collapses with high-quality sequencing data because there are so many pairwise combinations to be tested that statistical noise overcomes the genetic signal by orders of magnitude. So far, the second strategy is thus unavoidable for estimating epistasis from high-throughput sequencing data. On the one hand, some epistatic loci will not be detected (in particular, those involved in sign epistasis, which may have no marginal effect). On the other hand, we know from Equation (2) that the impact of loci on the composite epistatic coefficient is weighted by their (marginal) genetic variance, meaning that the loci with no additive effects will not affect directional epistasis. Consequently, estimating epistatic noise in general remains a complex task, and may require further statistical development. When it comes to directional epistasis, focusing on major loci is much less problematic and ensures a proper estimation of this biologically meaningful parameter.

### 4.3. Consistency across estimates

This paper illustrates the estimation of epistasis directionality by several methods, using independent data describing the same biological system. The various estimates are reported in Table 2. The units and the meaning of the epistatic coefficients differ according to the method. In order to facilitate the comparison, an epistatic factor *f*_100_ is provided. This factor corresponds to the coefficient by which genetic effects change when body weight increases by (arbitrarily) 100 g.

**Table 2:** **Summary of the directional epistasis estimates from different sources of data and different methods**.

**Source of data**	**Method**	**Estimate**	***f*_100_**
Selection response	Quadratic approximation (Equation 3)	ε = 6.6 × 10^−5^ g^−1^	1.007
Line cross	Line cross analysis (Equation 4)	ε = 1.9 × 10^−3^ g^−1^	1.19
QTL	Multilinear regression	ε = 5.7 × 10^−2^ g^−1^	6.7
QTL	Power model (Equation 7)	β = 2.18	6.6
QTL	Quadratic model	β′ = 8.3 g	2.0

Estimates can be compared with the *f*_100_ factor.

Directional epistasis estimates are consistently positive, and in most cases statistically significant. This provides strong confirmation that the genetic architecture of the weight differences between the high and low chicken lines is characterized by positive epistasis. However, the epistatic coefficients vary by several orders of magnitude in the different experiments; two categories of estimates can be defined: epistasis is strong when measured from the genotype data (increasing the phenotype by 100 g multiplies the allelic effects by 2 to almost 7), but weaker when measured from phenotype data (increasing the phenotype by 100 g increases allelic effects by 0.7 to 19%).

**Figure 7:**
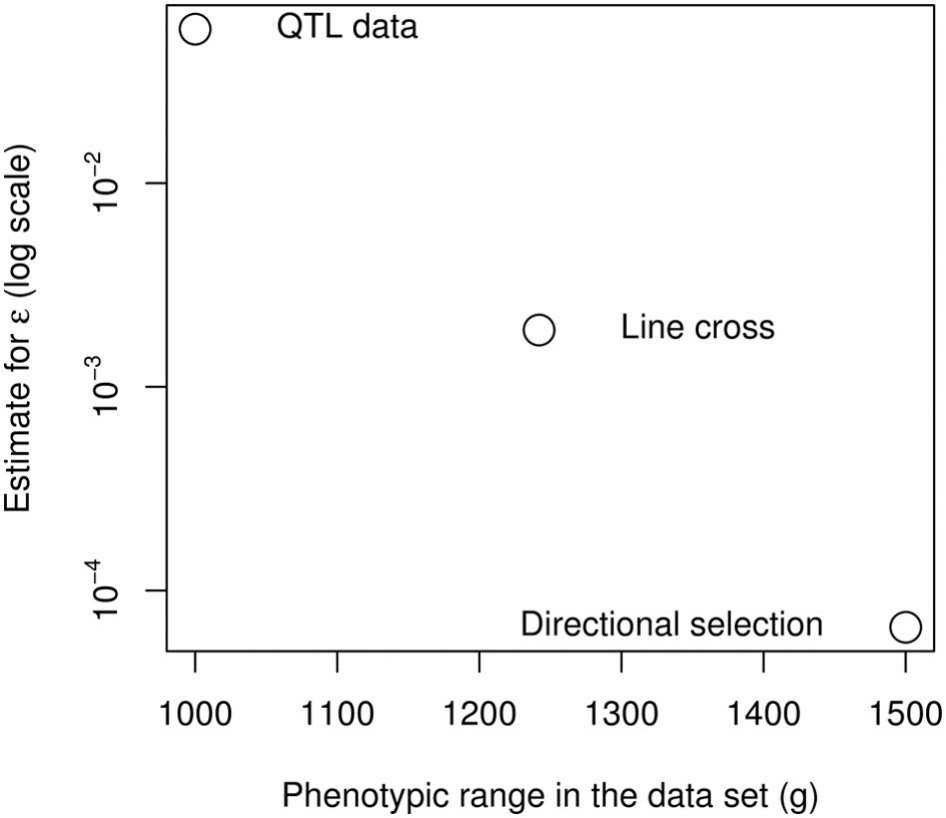
**Negative relationship between the span of the phenotypes in the data set and the directional epistasis coefficient**.

These measures are not necessarily contradictory, because epistasis can be restricted to a specific subset of the genetic architecture. As the epistatic coefficient measures the “average” curvature of the genotype-phenotype map, it is strongly affected by the nature of the data (and more specifically, the span of the data in terms of number of loci and phenotype range), as it seems to be the case for the chicken bodyweight (Figure 7). The extreme epistatic factors measured from the QTL data can be attributed to several factors. The four large-effect QTLs are not a random sample of loci, their effect is statistically inflated by detection bias (the Beavis effect: Beavis, 1994; Xu, 2003), and their strong epistatic interactions remain atypical (Carlborg et al., 2006). Their interaction pattern involves sign epistasis (Le Rouzic et al., 2007), so that additive effects vanish in some genetic backgrounds: increasing a small effect by a large factor does not necessarily mean that the absolute interaction effect is huge. In any case, even if positive epistasis is very strong for the 4 major loci, these QTLs only explain 7% of the total phenotypic variance, and the F_2_ population covers only 50% of the phenotype range of the parental lines. If directional epistasis is not a property of the whole genetic architecture, but merely reflects specific interactions between a few loci, data involving more loci and more genetic backgrounds would be expected to reveal less directional epistasis, which seems to be the case here with a striking regularity among the three independent data sources (Figure 7).

## 5. Concluding remarks

Unidimensional directional epistasis measures how the properties of genetic architectures change with the phenotype. It has often been confused with scaling. Scale transformation is a common operation in biology, often motivated by the need to make the data suitable for a particular statistical analysis (e.g., enforcing normality). Changing the scale of the phenotype measurement impacts on directional epistasis (Pavlicev et al., 2010), and it is possible to find an arbitrary scale transformation on which directional epistasis becomes negligible (or even is canceled out) in a data set. Applying such *ad hoc* mathematical operations to phenotypes prior to analysis could hardly be considered good practice. First, it has been repeatedly pointed out to biologists that, according to measurement theory, scales do actually have a meaning, and are thus not interchangeable (Wagner et al., 1998; Houle et al., 2011). One of the best examples is fitness, which is essentially multiplicative (Wagner, 2010). Epistasis on fitness thus has to be measured as the deviation from log-linearity, which justifies models of directional epistasis presented above. Obviously, directional epistasis following the power model cancels out on a log scale, but such a double log transformation would be meaningless, and should not be seriously considered. A second reason why scale change does not solve the problem of directional epistasis is that one should not necessarily expect consistent directionality. As exemplified by the chicken example, and illustrated in Figure 2, directionality is a local measure of the interlocus curvature of the genotype-phenotype map. It is thus likely that directionality could itself evolve as the phenotype changes (in the presence of third-order epistasis and higher-order interactions, directionality could even change when the phenotype remains constant). Therefore, comparing the properties of genetic architectures across populations or species requires measuring directional epistasis on a common scale.

Recent conceptual and theoretical advances have convincingly demonstrated that what matters in epistasis is not its direct contribution to genetic variation (interaction variance), but rather its propensity to (indirectly) influence the evolution of additive genetic variance. This propensity can be estimated by looking for specific patterns among epistatic interactions. The directionality of epistasis may be the most obvious, but other patterns are also emerging as candidate contributors to the evolvability of genetic architectures, such as the monotonicity of the genotype-phenotype relationship (closely linked to sign epistasis) (Gjuvsland et al., 2011, 2013), and the robustness or canalization of genetic architectures (Hermisson and Wagner, 2004; Draghi et al., 2010; Fraser and Schadt, 2010; Le Rouzic et al., 2013).

In quantitative genetics and breeding, correctly describing epistasis can improve the prediction of selection responses. In evolutionary genetics, epistasis determines the structure of genetic diversity and variability. At the phylogenetic scale, directional epistasis could contribute to biased anagenesis patterns and affect evolutionary trajectories. Most molecular mechanisms do not simply add up, and the genotype-phenotype relationship has to be curved to some extent. Is the observed curvature (quantified with one or several of the methods described here) consistent with predictions from system-biology models? To what extent is it constrained by the physical properties of the phenotypic trait? Does it vary depending on the trait, on the species? Does it evolve rapidly? The importance of determining directional epistasis for a wide diversity of traits in many organisms has probably been underestimated in the past, but now appears to be a key toward obtaining a better understanding of the general properties of genetic architectures.

### Conflict of interest statement

The Guest Associate Editor, Dr. José M. Alvarez-Castro, declares that, despite having collaborated on a publication with the authors in the last 2 years, the review process was handled objectively. The author declares that the research was conducted in the absence of any commercial or financial relationships that could be construed as a potential conflict of interest.

## Appendix I: multilinear epistasis on a continuous genotype-phenotype map

### Two loci

The multilinear model of Hansen and Wagner (2001b) is defined based on a reference genotype, and proposes a change-of-reference operation to recompute the genetic effects in a different genotype, assuming a multilinear genotype-phenotype map. In an arbitrary genotype-phenotype relationship, the multilinear model can be considered to be a local approximation of the multilocus curvature, and epistatic coefficients can be calculated from Taylor polynomial coefficients.

Let *g*(*y*_1_, *y*_2_) be a continuous and differentiable (at least twice) two-dimensional Genotype-Phenotype function associating a phenotype value *P* to any genotype combination (*y*_1_, *y*_2_) at two loci. The gradient vector at a particular genotype Γ = (Γ_1_, Γ_2_) is **D** (*D*_*i*_ = ∂ *g*(*y*_1_, *y*_2_) / ∂ *y*_*i*_ |_Γ_1_, Γ_2__), and the Hessian matrix is **D**^2^ (*D*^2^_*i*, *j*_ = ∂^2^ *g*(*y*_1_, *y*_2_) / ∂ *y*_*i*_ ∂ *y*_*j*_ |_Γ_1_, Γ_2__). The second-order Tailor series around this genotype Γ is:

(A1) $$\begin{array}{l} P(y_{1},y_{2})≃g(\Gamma_{1},\Gamma_{2})+D_{1}(y_{1}-\Gamma_{1})+D_{2}(y_{2}-\Gamma_{2})\\ \;+\frac{1}{2}D_{1,1}^{2}{\left(y_{1}-\Gamma_{1}\right)}^{2}+\frac{1}{2}D_{2,2}^{2}{\left(y_{2}-\Gamma_{2}\right)}^{2}\\ \;+D_{1,2}^{2}(y_{1}-\Gamma_{1})(y_{2}-\Gamma_{2}). \end{array}$$

Rescaling as *y*′_1_ = *D*_1_(*y*_1_ − Γ_1_) and *y*′_2_ = *D*_2_(*y*_2_ − Γ_2_) and neglecting the quadratic terms leads to a multilinear approximation taking the genotype Γ as a reference point:

(A2) $$P(y_{1}^{\prime },y_{2}^{\prime })≃g(\Gamma_{1},\Gamma_{2})+y_{1}^{\prime }+y_{2}^{\prime }+y_{1}^{\prime }y_{2}^{\prime }\frac{D_{1,2}^{2}}{D_{1}D_{2}},$$

where it appears clearly that the directionality coefficient of Hansen and Wagner (2001b) is ε_*ij*_ = *D*^2^_*i*, *j*_/*D*_*i*_ *D*_*j*_. The quadratic terms $\frac{1}{2}$ *D*^2^_1, 1_ *y*′^2^_1_ and $\frac{1}{2}$ *D*^2^_2, 2_ *y*′^2^_2_ disappear from the equation as a consequence of the multilinear approximation.

### Several loci

The previous approximation can be extended to several loci in a straightforward way:

(A3) $$\varepsilon_{ij}=\frac{\partial^{2}g}{\partial y_{i}\partial y_{j}}{|}_{\Gamma }/\frac{\partial g}{\partial y_{i}}{|}_{\Gamma }\frac{\partial g}{\partial y_{j}}{|}_{\Gamma }.$$

Developing the third-order Taylor series and neglecting all quadratic terms, the third-order epistatic coefficients can be written as follows:

(A4) $$\varepsilon_{ijk}=\frac{\partial^{3}g}{\partial y_{i}\partial y_{j}\partial y_{k}}{|}_{\Gamma }/\frac{\partial g}{\partial y_{i}}{|}_{\Gamma }\frac{\partial g}{\partial y_{j}}{|}_{\Gamma }\frac{\partial g}{\partial y_{k}}{|}_{\Gamma }.$$

The multilinear approximation can thus be easily extended to any number of loci and any order of epistasis, with the *n*^th^ order epistasis coefficient being the *n*^th^ mixed partial derivative of the genotype-phenotype function scaled by the product of the first-order derivatives of this function for all loci involved in the interaction.

## Appendix II: effect of directional epistasis on artificial selection response

The impact of directional epistasis on the response to directional selection is rather complex to predict precisely for arbitrary time periods (Carter et al., 2005). Nevertheless, useful approximations can still be derived by making realistic assumptions about the properties of genetic architectures. For instance, Le Rouzic et al. (2011) proposed a model that can be simplified as:

(A5a) $$\mu_{t\;+\;1}\;=\mu_{t}+V_{A_{t}}\beta_{t}$$

(A5b) $$V_{A_{t+1}}\;=V_{A_{t}}+2\beta_{t}\varepsilon V_{A_{t}}^{2}$$

Equation (A5a) is the traditional breeder's equation, formulated as in Lande and Arnold (1983), where *V*_*A*_ is the additive genetic variance, and β the selection gradient, i.e., the slope of the regression between phenotype and relative fitness. Equation (A5b) approximates the impact of directional epistasis on additive variance, summarized by the directionality coefficient ε.

This model requires 3 parameters: μ_0_, the initial phenotype, the initial additive variance *V*_*A*_0__, and the epistatic parameter ε. Fitting the model by maximizing its likelihood for phenotype times series including means and variances provide convincing estimates of epistasis, especially when the data include bidirectional artificial selection (Le Rouzic et al., 2011).

Unfortunately, variance time series are not always available from historical data, because either they were measured but not reported in the corresponding publications, or simply because they were not computed, as only the mean phenotype was the center of interest. Moreover, fitting such a complex multidimensional non-linear model can be tricky, and requires significant computer programming input (and possibly having to solve numerical convergence issues). Proposing simpler formulas could therefore be helpful, as they may allow any biologist with basic statistical knowledge to report the strength of directional epistasis based on average phenotype data.

The following calculation is based on several approximations, the main ones being that selection is expected to be constant (β_*t*_ = β), and that linkage disequilibrium can be ignored. If directional epistasis is the only phenomenon affecting the selection response, the additive genetic variance is expected to change as in Equation (A5b). Approximating the discrete process by a continuous function leads to the ordinary differential equation $\frac{\text{d}V_{A}}{\text{d}t}$ = 2βε *V*^2^_*A*_, which can be solved as:

(A6) $$V_{A_{t}}=\frac{V_{A_{0}}}{1-2\beta V_{A_{0}}\varepsilon t}.$$

Assuming that directional epistasis is not very strong (εβ *V*_*A*_0__ ≪ 1), the expected phenotype at time *t* results from the product between the (supposedly constant) selection gradient β and the cumulative change in *V*_*A*_, which can be calculated as:

(A7) $$\mu_{t}=\mu_{0}+\beta \int_{0}^{t}V_{A_{\tau }}\text{d}\tau =\mu_{0}-\frac{\log(1-2\beta V_{A_{0}}\varepsilon t)}{2\varepsilon }.$$
