# Corrigendum for “Dissecting genetic effects with imprinting”

**DOI:** 10.3389/fgene.2014.00427

**Published:** 2014-12-08

**Authors:** José M. Álvarez-Castro

**Affiliations:** Department of Genetics, Universidade de Santiago de CompostelaLugo, Spain

**Keywords:** imprinting, NOIA, individual-referenced models of genetic effects, population-referenced models of genetic effects, genetic variance decomposition

Corrigendum:

In the article of the Frontiers Research Topic Issue on Models and Estimation of Genetic Effects “Dissecting genetic effects with imprinting,” by Álvarez-Castro (2014), the citation of the work in press by Álvarez-Castro and Le Rouzic is no longer correct since its publication has in the end been postponed to 2015 (Álvarez-Castro and Le Rouzic, 2015). The same holds for the citation of that reference in press in the article “Estimating directional epistasis,” by Le Rouzic (2014), also within the Frontiers Research Topic Issue on Models and Estimation of Genetic Effects.

## Conflict of interest statement

The author declares that the research was conducted in the absence of any commercial or financial relationships that could be construed as a potential conflict of interest.
